# RASAL1 induces to downregulate the SCD1, leading to suppression of cell proliferation in colon cancer via LXRα/SREBP1c pathway

**DOI:** 10.1186/s40659-019-0268-x

**Published:** 2019-12-17

**Authors:** Guangchuan Wang, Zhen Li, Xiao Li, Chunqing Zhang, Lipan Peng

**Affiliations:** 1 Department of Gastroenterology and Hepatology, Shandong Provincial Hospital Affiliated to Shandong University, 324 JingwuWeiqi Road, Jinan, Shandong 250021 People’s Republic of China; 2 Department of General Surgery, Shandong Provincial Hospital Affiliated to Shandong University, 324 JingwuWeiqi Road, Jinan, Shandong 250021 People’s Republic of China

**Keywords:** RASAL1, Colon cancer, LXRα, SREBP1c, Fatty acid synthesis, SCD1

## Abstract

**Background:**

Recent studies have confirmed that RASAL1 has an antitumor effect in many cancers, but its functional role and the molecular mechanism underlying in colon cancer has not been investigated.

**Results:**

We collected human colon cancer tissues and adjacent non-tumor tissues, human colon cancer cell lines LoVo, CaCo2, SW1116, SW480 and HCT-116, and normal colonic mucosa cell line NCM460. RT-qPCR was used to detect the RASAL1 level in the clinical tissues and cell lines. In LoVo and HCT-116, RASAL1 was artificially overexpressed. Cell viability and proliferation were measured using CCK-8 assays, and cell cycle was detected via PI staining and flow cytometry analysis. RASAL1 significantly inhibited the cell proliferation via inducing cell cycle arrest, suppressed cell cycle associated protein expression, and decreased the lipid content and inhibited the SCD1 expression. Moreover, SCD1 overexpression induced and downregulation repressed cell proliferation by causing cell cycle arrest. Additionally, luciferase reporter assays were performed to confirm the direct binding between SREBP1c, LXRα and SCD1 promoter, we also demonstrated that RASAL1 inhibit SCD1 3′-UTR activity. RASAL1 inhibited tumor growth in xenograft nude mice models and shows inhibitory effect of SCD1 expression in vivo.

**Conclusion:**

Taken together, we concluded that RASAL1 inhibited colon cancer cell proliferation via modulating SCD1 activity through LXRα/SREBP1c pathway.

## Background

Colon cancer ranks third and fourth in the worldwide rankings of cancer incidence and mortality, respectively [1], with more than 1.3 million new cases reported annually and nearly 0.5 million deaths each year [2]. Multiple factors are involved in the occurrence and development of colon cancer, including the activation of oncogenes and inactivation of tumor suppressor genes [3]. Among them, the classical Ras guanosine triphosphate hydrolases (GTPases), H-Ras, K-Ras, and N-Ras, are critical regulators of proliferation, and it is thus not surprising that they represent the most frequently mutated human oncogenes [4]. The RAS GTPase-activating protein (Ras-GAP) gene RASAL1 has been demonstrated as a tumor suppressor gene which act as a negative modulator of the RAS signaling pathway by catalyzing RAS inactivation [5]. RASAL1 expression is decreased in many tumors, including thyroid cancer, gastric cancer, prostatic cancer and bladder cancer [5–8], but its functional role in colon cancer has not been investigated. The RAS oncogene family could encode membrane-associated proteins involved in mediation of signals arising from binding of ligands to cell membrane receptors such as epidermal growth factor receptors (EGFRs) to nuclear transcription factors [9].

Stearoyl-coenzyme A desaturase 1 (SCD1) is a central regulator of fuel metabolism and may represent a therapeutic target to control obesity and the progression of related metabolic diseases including type 2 diabetes and hepatic steatosis [10]. Rapidly-proliferating cancer cells often have a robust program of fatty acid synthesis, so the associated genes such as SCD1 was pursued as an oncology target [11]. SCD1 contributes to maintain a shift in lipid metabolism (increase in lipogenesis and inhibition of fatty acid oxidation) and intracellular signaling (activation of Akt signals and deactivation of the AMPK pathway), therefore favoring an accelerated rate of cell proliferation, increased invasiveness, enhanced survival and, ultimately, a greater tumorigenic capacity [12]. And the SCD1 pharmacological inhibition or gene silencing resulted in decreased spheroid formation efficiency of primary lung cancer cells and induced a reduction in aldehyde dehydrogenase activity and in the expression of several other stemness markers, may as the potential development of novel anticancer therapies [13, 14].

Previous studies found that, LXRα activation increased hepatic fatty acid desaturation via the induction of SCD1 expression in an LXRα-dependent, SREBP1c-mediated manner [10]. The activated LXR showed synergy with EGFR antagonists, thus pointing out the cholesterol pathway as a critical target regulating the growth of human carcinomas [11]. LXRα activation increased SCD1 protein level through upregulate its target gene SREBP-1 and its consequent binding to SRE1 sequence in HepG2 cells [12]. Until now, rarely studies have focused on the relationship between RASAL1 and the fatty acid synthesis associated gene. Since Ras as the upstream in the regulating of SREBP1c expression [15], RASAL1 as the RAS GTPase-activating protein, so we suggest whether RASAL regulate the SCD1 activity also via LXRα and SREBP-1 pathway in colon cancer.

In this study, an decreased in RASAL1 were identified in colon cancer tissue. In addition, our also found that, RASAL1 could facilitate the proliferation and fatty acid synthesis of colon cancer cell lines. These results for the first time identified the role of RASAL1 in colon cancer was associated with decreased SCD1, which was demonstrated to be a novel target gene of RASAL1 and implications for improving the treatment of colon cancer.

## Materials and methods

### Colon cancer tissue samples

The 27 samples of colon cancer and adjacent non-tumor tissues were collected from patients diagnosed in our hospital from August 2017 to September 2018. All patients who provided samples had been diagnosed in pathological examinations and not treated with radiotherapy and chemotherapy before surgery. Informed consent was obtained from each patient, and all the experiments were approved by the Ethics Committee of Shandong University. All tissue samples were maintained in liquid nitrogen.

### Cell lines and cell culture

The human colonic carcinoma cell lines LoVo, CaCo2, SW116, SW480 and HCT-116, and the normal colonic mucosa cell line NCM460 were purchased from the Shanghai Cell Bank, China. All cell lines were cultured in DMEM medium containing 10% FBS, 100 U/ml penicillin and 100 mg/ml streptomycin at 37 °C in a 5% CO_2_ incubator. A939572 (#HY-50709), T0901317 (#HY-10626) both were purchased from MedChem Express, Shanghai, China.

### RNA extraction and quantitative RT-qPCR analysis

Total RNA was extracted from tissues or cells using RNAiso Plus (Takara Bio Inc., Dalian, China), DNaseI-treated RNA was used for first strand cDNA synthesis using MMLV reverse transcriptase (Promega Corporation, Madison, WI, USA) and oligo(dT)_15_ according to the manufacturer’s protocol and 1 μL cDNA samples were used for conventional PCR amplifications. SYBR Premix Master Mix (Thermo Scientific Inc., Waltham, MA, USA) was used to conduct real-time PCR with Bio-Rad IQ-5 with the reaction consisting of a 10-min hot start at 95 °C, then 40 cycles of 15 s at 95 °C, 30 s at 60 °C, and 30 s at 72 °C. The 2^−ΔΔCt^ method was used for mRNA quantification analysis, with 18S as standards. The primer sequences used in the experiments were as follows:

- Homo-Ppar γ forward 5′-GGGATCAGCTCCGTGGATCT-3′, reverse 5′-TGCACTTTGGTACTCTTGAAGTT-3′;
- Homo-Srebp1c forward 5′-GCCCCTGTAACGACCACTG-3′, reverse 5′-CAGCGAGTCTGCCTTGATG-3′;
- Homo-acly forward 5′-ATCGGTTCAAGTATGCTCGGG-3′, reverse 5′-ACCAAGTTTTCCACGACGTT-3′;
- Homo-Fasn forward 5′-AAGGACCTGTCTAGGTTTGATGC-3′, reverse 5′-TGGCTTCATAGGTGACTTCCA-3′;
- Homo-Acc1 forward 5′-ATGTCTGGCTTGCACCTAGTA-3′, reverse 5′-CCCCAAAGCGAGTAACAAATTCT-3′;
- Homo-Acc2 forward 5′-CAAGCCGATCACCAAGAGTAAA-3′, reverse 5′-CCCTGAGTTATCAGAGGCTGG-3′;
- Homo-Dgat1 forward 5′-CAATCTGACCTACCGCGATCT-3′, reverse 5′-TCGATGATGCGTGAGTAGTCC-3′;
- Homo-Dgat2 forward 5′-TCAGCTTGGGTACGAAACTGG-3′, reverse 5′-GGGGTGATTGGCAATGATGTAG-3′;
- Homo-Mttp forward 5′-ATTGTAAAGTGACCTACCAGGCT-3′, reverse 5′-ACCTCGCTATTTTGCATGAATCC-3′;
- Homo-Apob forward 5′-TGCTCCACTCACTTTACCGTC-3′, reverse 5′-TAGCGTCCAGTGTGTACTGAC-3′;
- Homo-Vldlr forward 5′-CTGTGTAAAGAAGACGTGTGCT-3′, reverse 5′-TGATTTCATGTATGCGGCATGT-3′;
- Homo-Rasal1 forward 5′-CAGCTCCCTGAATGTTCGC-3′, reverse 5′-TCCTCATCCAGCACGTAGAAG-3′;
- Homo-Scd1 forward 5′-TCTAGCTCCTATACCACCACCA-3′, reverse 5′-TCGTCTCCAACTTATCTCCTCC-3′;
- Homo-GAPDH forward 5′-TGTGGGCATCAATGGATTTGG-3′, reverse 5′-ACACCATGTATTCCGGGTCAAT-3′.

### Plasmid and transfection

R777-E233 Hs.RASAL1 was a gift from Dominic Esposito (Addgene plasmid # 70517; http://n2t.net/addgene:70517; RRID:Addgene_70517), and subcloned into pcDNA-3HA. Complementary DNA for human SCD1 was cloned into pcDNA-3HA plasmid to obtain SCD1-HA tagged protein. As predicted from the JASPAR database (http://jaspar.genereg.net/), RASAL1 can bind on − 710 to − 691 of SCD1 promoter. The point-mutation in the RASAL1 binding site of SCD1 promoter was generated by site-directed mutagenesis that splices by overlap extension with Mut Express MultiS Fast Mutagenesis Kit V2 (#C215-01, Vazyme, Nanjing, China). Colon cancer cells were seeded in 6-well plates overnight (1 × 10^6^ per well). Cells were transfected with vector control and RASAL1, SCD1 or vector control using Lipofectamine 2000 (Invitrogen) according to the manufacturers’ instructions, 6 h after transfection, the cells were cultured in normal culture medium. Analyses were performed 48 h later.

### Nile red staining

HCT-116 cells were transfected with RASAL1 or vector control for 24 h, and treated with 100 μM Oleic acid (OA) for another 24 h. After treatment, cells were washed with PBS and fixed in a methanol: acetic acid (3:1) solution overnight at 4 °C. Following it, cells were washed with PBS and incubated in 500 ng/mL Nile red (Sigma–Aldrich, St. Louis, MO) in PBS for 30 min at 37 °C. Analysis was carried on BD FACS Aria II Flow Cytometer (Becton–Dickinson, Franklin Lakes, NJ, USA) [16]. Gating was done in SSC-A versus FSC-A graph to avoid artifacts due to noise or cell debris.

### Dual-luciferase reporter assay

A Dual-luciferase reporter assay was performed to explore whether SCD1 was a target of RASAL1. Cells were transfected with vector control or RASAL1 and cotransfected with pGL3-SCD1-3′UTR wild-type or pGL3-SCD1-3′UTR mutant using Lipofectamine 3000, then treated with 1 μM A939572 or 1 μM T0901317. Following incubation for 48 h, a Dual-luciferase reporter assay (Promega Corporation, Madison, WI, USA) was performed to detect firefly and *Renilla* luciferase activity according to the manufacturer’s protocol. *Renilla* luciferase activity was measured as an internal control. Each experiment was repeated at least three times.

### Cell cycle analysis

Cells were plated at a density of 1 × 10^6^ cells/ml in each well of 6 well plates followed by transfected with vector, RASAL1 or SCD1 for 48 h. After fixing with 70% ethanol for 30 min, cells were incubated with 50 μg/ml propidium iodide (Fisher scientific, Pittsburgh, PA) and 100 μg/ml RNase (Fisher Scientific, Pittsburgh, PA) at room temperature in the dark for 15 min. Cells were analyzed by Flow cytometer. The particular phase of the cell cycle with DNA content in G0/G1, S and G2/M was estimated using FlowJo software v 10.2.

### Western blot

Total lysates from tissues or cells were obtained by lysing in RIPA buffer with protease inhibitors cocktail (#HY-K0010, MedChem Express, Shanghai, China). Protein concentration was measured by the BCA assay (Bio-Rad, Hercules, CA, USA). Proteins were extracted and separated in 10% Tris glycine/SDS–polyacrylamide gels and transferred to PVDF membranes (#IPFL00010, Millipore, Bedford, MA, USA). The membranes were blocked with 5% nonfat milk and incubated with specific antibodies overnight at 4 °C. β-actin (Proteintech, #66009-1-Ig) was used as the endogenous control. Primary antibodies were used at the dilution of 1:1000. Anti-SCD1 (#2794), cyclin D1 (#2978), cyclin D2 (#3741), cyclin D3 (#2936), cyclin E1 (#4129), CDK4 (#12790), CDK6 (#13331), CDK2 (#2546), P18 (#2896), P21 (#2947), P27 (#3686), P-Rb (#8516) were purchased from Cell Signaling Technology (Beverly, MA, USA). Anti-RASAL1 (ab168610) was obtained from Abcam (Cambrdige, MA, USA). Blots were then incubated with relevant secondary antibodies, HRP-conjugated Goat Anti-Rabbit IgG (SA00001-2) and HRP-conjugated Goat Anti-mouse IgG (SA00001-1) were purchased from Proteintech (Wuhan, China) for 1 h. Bands were detected with the enhanced chemiluminescence detection systeme (P10200, New Cell & Molecular Biotech Co., Ltd) and Bio-Rad ChemiDocTM MP imaging system. Relative abundance was measured with Image J software.

### Nude mice model

The animal experiments were approved by the Committee on Animal Care of Shandong University and were conducted according to NIH Guidelines for the Care and Use of Laboratory Animals. All studies involving animals are reported in accordance with the ARRIVE guidelines for reporting experiments involving animals. Twenty SPF male Balb/c nude mice aged 5–6 weeks and weighing 18–20 g were purchased from the Vital River Laboratories (Beijing, China) and divided into two groups randomly. A total of 6 × 10^6^ HCT-116 cells which were stably transfected with vector control or RASAL1 were injected subcutaneously into the left flank of nude mice. Tumor volumes were calculated using the formula V = length × width^2^/2. The animals were sacrificed 4 weeks after injection. Pictures were recorded with a digital camera.

### Statistical analysis

The data were expressed as the mean ± standard error of mean (SEM) and analyzed using the SPSS 20.0 statistical software (SPSS Inc., USA). The comparisons between groups were analyzed using ANOVA followed by least-significant difference post hoc analysis, and P < 0.05 was considered statistically significant.

## Results

### RASAL1 is expressed at a low level in colon cancer

**Fig. 1:**
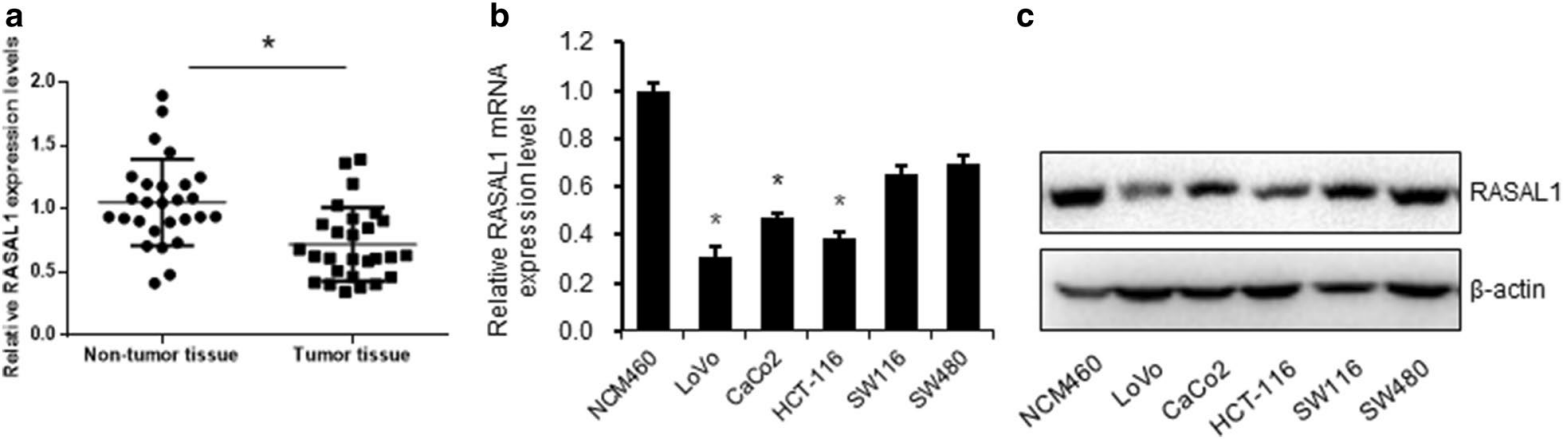
RASAL1 is downregulated in colon cancer samples and cell lines. **a** Expression of RASAL1 in 27 pairs of colon cancer samples was compared with that in adjacent non-tumor tissues; determination via quantitative RT-qPCR. **b** Quantitative RT-qPCR and **c** Western blot were performed to detect the expression of RASAL1 in 5 colon cancer cell lines (LoVo, CaCo2, HCT-116, SW1116 and SW480) and a normal colonic cell line (NCM460). *P < 0.05

In order to understand the role of RASAL1 during the progress of colon cancer, we first determined the expression levels of RASAL1 in 27 pairs of colon cancer tissues and adjacent normal tissues using quantitative RT-qPCR. As shown in Fig. 1a, RASAL1 has a low level of expression in colon cancer tissues. Next, we detected the expression level of RASAL1 in colon cancer cell lines. Compared to the mRNA and protein expression levels in the normal colonic epithelial cell line NCM460, RASAL1 has a low level of expression in multiple colon cancer cell lines, including LoVo, CaCo2, HCT-116, SW116 and SW480 (Fig. 1b, c). As LoVo and HCT-116 shows the relative lower RASAL1 expression levels than the other three cell lines, these two cells will be used in the further study.

### RASAL1 inhibited colon cancer cell proliferation accompanied with the alteration of SCD-1 expression

**Fig. 2:**
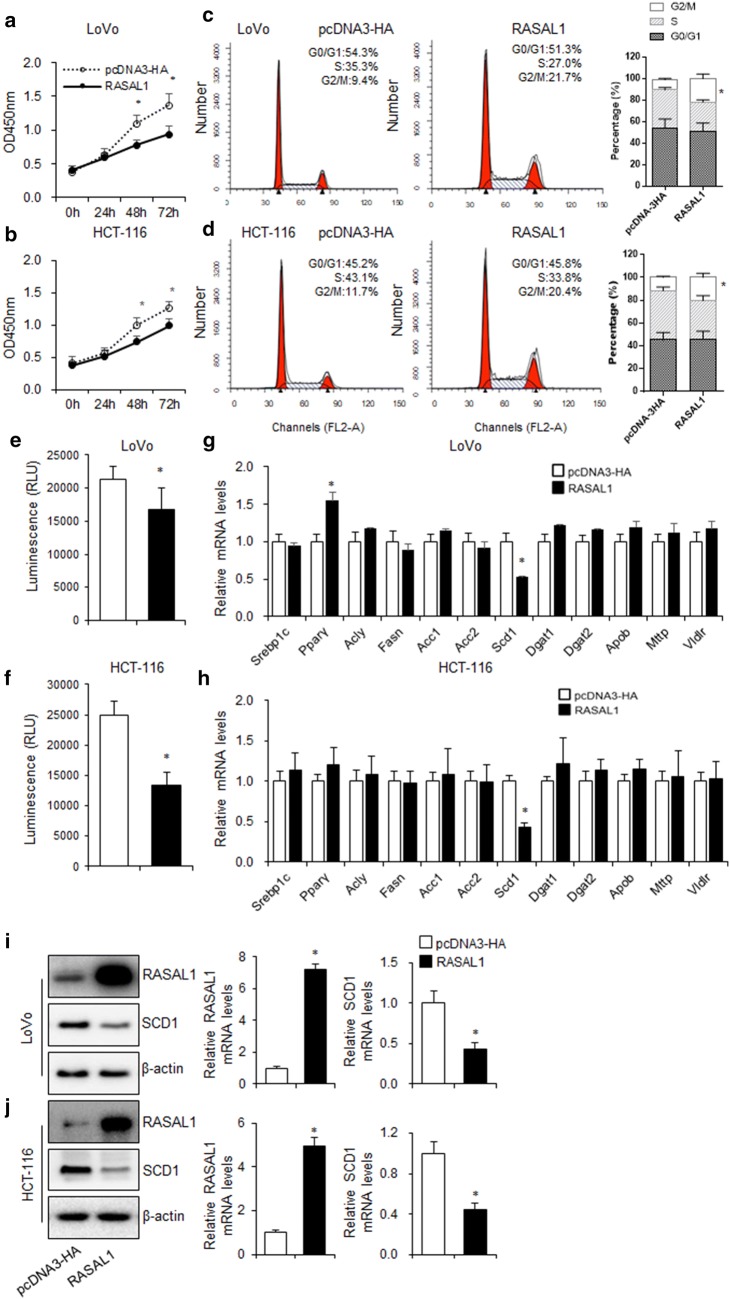
RASAL1 inhibited colon cancer cell proliferation accompanied by the inhibition of SCD1 expression. **a**–**d** LoVo and HCT-116 cells were transfected with RASAL1 for 48 h. The CCK-8 assay showed that RASAL1 inhibited the proliferation of **a** LoVo and **c** HCT-116 cells. Absorbance at 450 nm was measured at 24, 48, and 72 h. Data are expressed as mean ± SEM, n = 3, *P < 0.05. Cell cycle of **b** LoVo and **d** HCT-116 were analyzed by PI staining. Data are expressed as mean ± SEM, n = 3, *P < 0.05. **e**–**h** LoVo and HCT-116 cells were transfected with vector control and RASAL1 for 48 h, **e**, **g** followed by nile red staining, the lipid content was analyzed by flow cytometer, **f**, **h** RT-qPCR was used to detect the fatty acid synthesis related genes expression. **i**, **j** Western blotting and RT-qPCR assay were used to detect the SCD1 protein and mRNA expression levels in LoVo and HCT-116 cells transfected with RASAL1 for 48 h. Data are expressed as mean ± SEM, n = 3, *P < 0.05

To determine the effects of RASAL1 on the proliferation of colon cancer cells, CCK-8 assay was performed on LoVo and HCT-116 cells with RASAL1 in low levels. The results showed that the proliferation of colon cancer cells was significantly inhibited by RASAL1 overexpression (Fig. 2a, b). To investigate the mechanism responsible for the growth inhibition caused by RASAL1, cell cycle distribution was determined by flow cytometry analysis. After RASAL1 transfection for 24 h, the percentage of colon cancer cells in the G2/M phase increased (LoVo: 9.4–21.7%; HCT-116: 11.7–20.4%) compared to the control (Fig. 2c, d).

Cancer tissues could generate fatty acids (FAs) and phospholipids through cellular de novo lipogenesis, and were not solely reliant upon lipid/FA uptake from the environment. This is turn provided the support required for the excessive growth and proliferation, which is a hallmark of cancer [17]. To explore whether RASAL1 affect the fatty acid synthetic pathways in LoVo and HCT-116 cells, Neil Red staining was used and found that RASAL1 significantly decreased the lipid levels, confirming its ability to block energy metabolism in colon cancer cells (Fig. 2e, f). To identify key regulators A939 that may lower TAG content, we examined the expression of genes that regulate lipogenesis in RASAL1 overexpressed LoVo and HCT-116 cells. As shown in Fig. 2g, h, mRNA levels of most of the genes responsible for lipogenesis were unchanged, but the expression of SCD1 was down-regulated. This lipogenesis inhibition may be responsible for suppression of cell proliferation. Moreover, we found that the expression of SCD1 was obviously downregulated when the colon cancer cells were transfected with RASAL1 (Fig. 2i, j).

### Overexpression of SCD1 promoted colon cancer cell proliferation

**Fig. 3:**
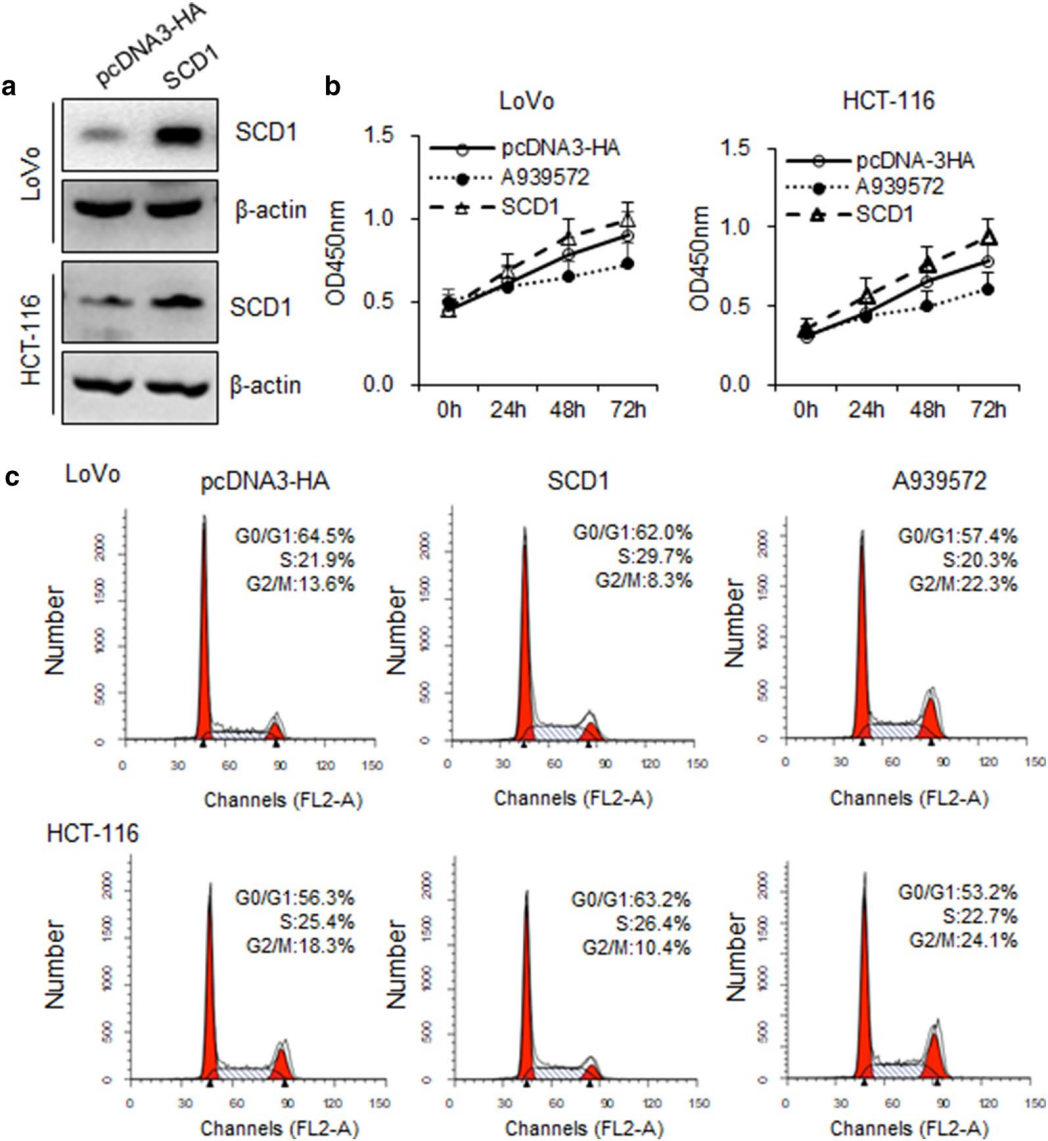
Overexpression of SCD1 inhibited colon cancer cells proliferation in vitro. **a** LoVo and HCT-116 were transfected with vector control and SCD1 for 48 h, and detected the transfection efficacy by western blot. Data are expressed as mean ± SEM, n = 3, *P < 0.05. **b** Growth curves of LoVo and HCT-116 cells after transfection with SCD1 and vector control were determined by CCK-8 assays. Data are expressed as mean ± SEM, n = 3, *P < 0.05. **c** Cell cycle assay showed that SCD1 caused G2/M arrest of LoVo (8.7–20.4%) and HCT-116 (6.5–17.9%) cells. Data are expressed as mean ± SEM, n = 3, *P < 0.05

To explore the function of SCD1 in the regulation of cell proliferation, 1 μM A939572 was used to inhibit the SCD1 enzymatic activity, and SCD1 expression vector was transfected into LoVo and HCT-116. The SCD1 expression was upregulated at 48 h compared to the vector control, determined by western blot (Fig. 3a). The effect of SCD1 expression on cell proliferation was evaluated by CCK-8 assay. Overexpression of SCD1 reduced the proliferation capacity of LoVo and HCT-116 cells, as compared with those transfected with vector control (Fig. 3b). To investigate the mechanism responsible for the growth inhibition caused by SCD1, cell cycle distribution was determined by flow cytometry analysis. After SCD1 transfection for 48 h, the percentage of colon cancer cells in the G2/M phase decreased (LoVo: 13.6%–8.3%; HCT-116: 18.3%–10.4%) compared to the control (Fig. 3c), 1 μM A939572 treatment for 48 h significantly increased the G2/M phase into 22.3% (LoVo) and 24.1% (HCT-116). Indicating that the repression of cell proliferation may due to cell cycle arrest.

### RASAL1 inhibits SCD1 activity via LXRα/SREBP1c pathway

**Fig. 4:**
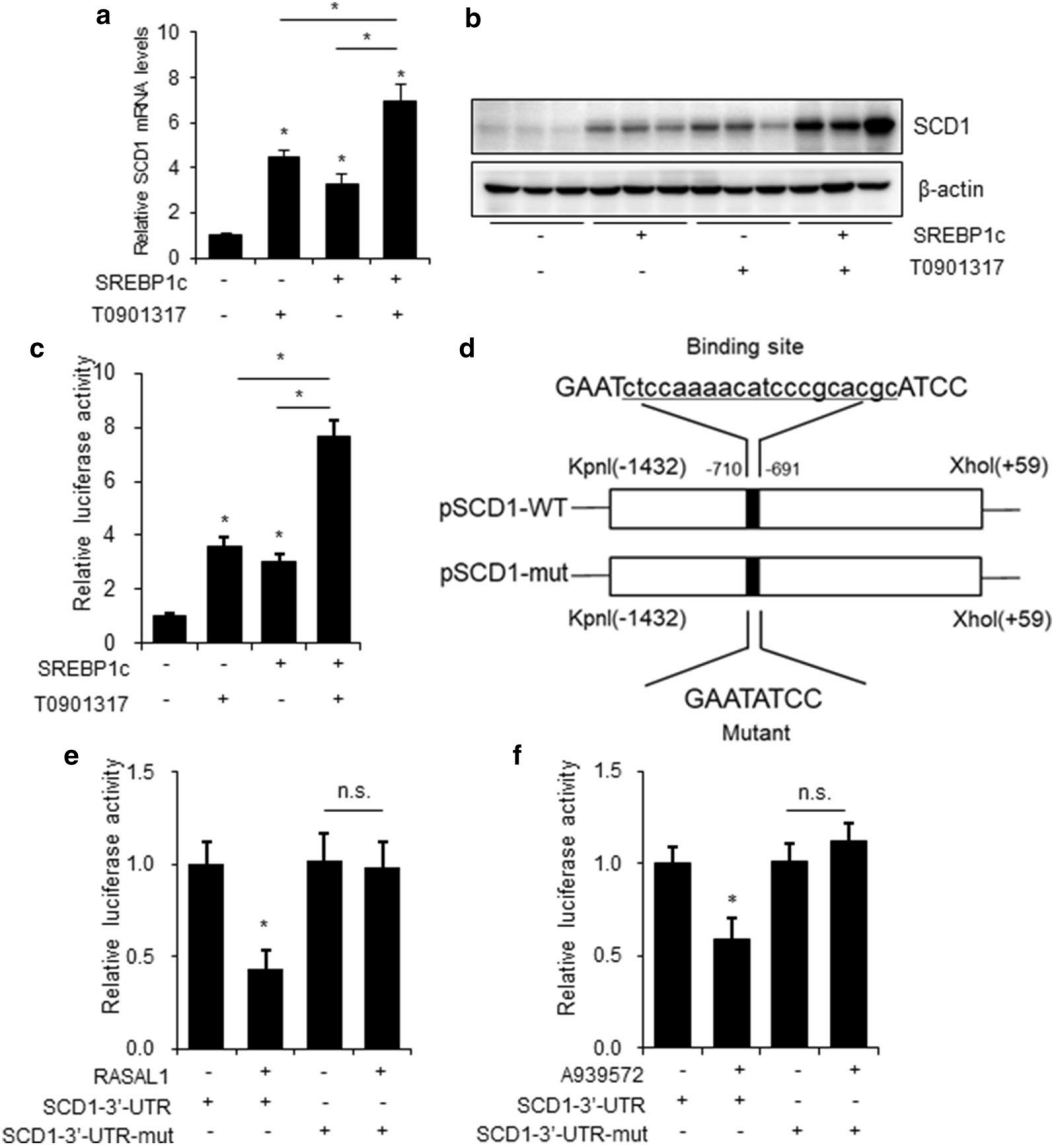
RASAL1 inhibited the activity of SCD1 3′-UTR through LXRα and SREBP1c. **a**, **b** SCD1 mRNA and protein levels were detected by RT-qPCR and western blot at 48 h after transfection with pcDNA3.1 or SREBP1c, or treated with 1 μM T0901317, β-actin was used as loading control. **c** Luciferase assay of HCT-116 cells co-transfected with the luci-SCD1 3′-UTR reporter with SREBP1c or treated with 1 μM T0901317. After 48 h, firefly luciferase values were normalized to β-gal luciferase activity. **d** Schematic illustration of point mutation of the binding site of RASAL1 and SCD1 promoter. **e**, **f** The effect of RASAL1 on luciferase intensity controlled by the wild type (WT) or mutant (Mut) 3′-UTR of SCD1 was determined using the luciferase assay. Each bar represents the mean ± SEM. The results were reproduced in three independent experiments. *P < 0.05

It is well established that SREBP1c has binding sites in the promoter region of the SCD1 gene and upregulates SCD1 transcription in liver, overexpression of SREBP1c or treat with LXRα activator T0901317 both upregulates the mRNA and protein levels of SCD1 in HepG2 cells [18]. So, we would like to test whether LXRα and SREBP1c has the same effect on HCT-116 cells. As shown in Fig. 4a, b, HCT-116 cell transfected with SREBP1c or treated with 1 μM T0901317 both up-regulated the SCD1 mRNA and protein levels. By co-transfecting RASAL1 with the SCD1–3′UTR reporter vector into HCT-116 cells, we detected the activity of luciferase to demonstrate the condition of RASAL1 targeting SCD1, the reporter activity was both induced by T0901317 treatment or SREBP1c overexpression independently, when both treated with T0901317 and SREBP1c, the SCD1 reporter activity was increased significantly (Fig. 4c). These results further support the possibility that LXRα and SREBP1c could induced SCD1 transcription in HCT-116 cells. At the same time, we made point mutations in SCD1–3′UTR to construct mutant SCD1–3′UTR fluorescence reporter plasmid (Fig. 4d). As shown in Fig. 4e, f, overexpression of RASAL1 resulted in decreased fluorescence activity and no significant changes in fluorescence activity were observed in mutant UTR, the SCD1 inhibitor showed the same effect.

### Overexpression of RASAL1 inhibited tumor growth in xenograft models of colon cancer

**Fig. 5:**
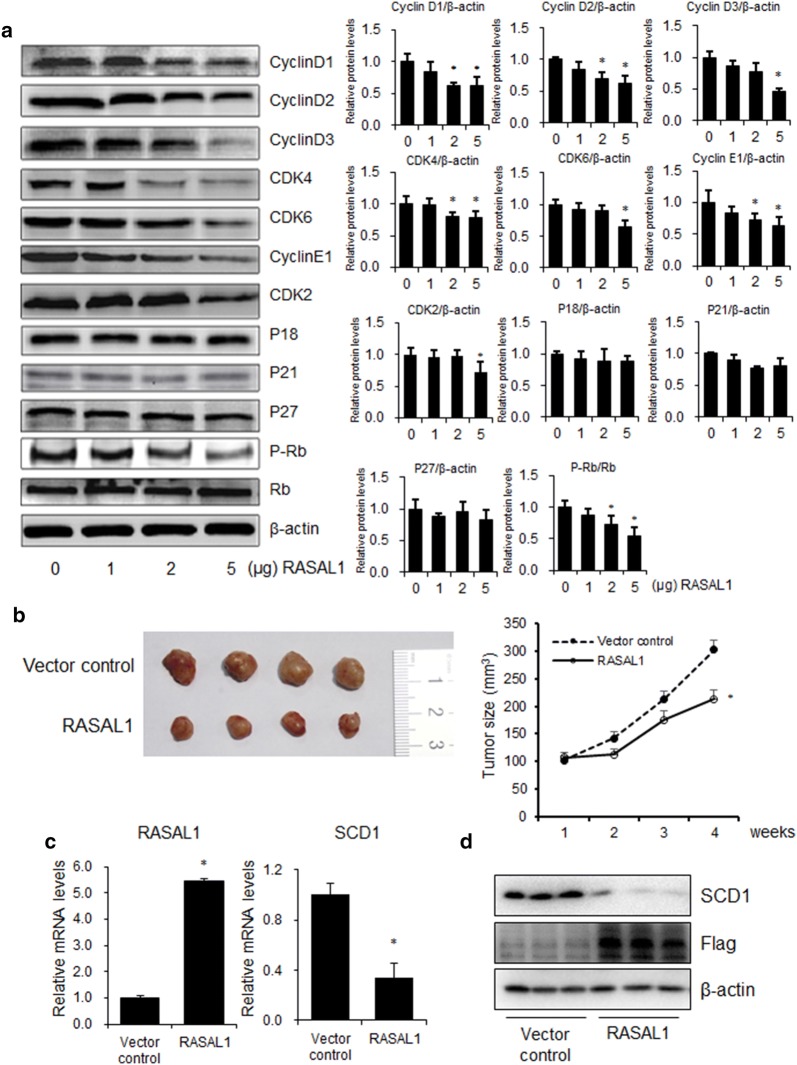
Overexpression of RASAL1 inhibited tumor growth in vivo. **a** HCT-116 cells were seeded into 6-well plate and transfected with vector control or 1 μg/well, 2 μg/well or 5 μg/well RASAL1 plasmid for 48 h, cell cycle related protein expression levels were detected using western blot. **b** HCT-116 cells were transfected with RASAL1 or control and injected subcutaneously into 12 nude mice in the flank region. Surgical resections of HCT-116 xenograft tumors on week 4 are shown. Measurements of tumor volumes were taken weekly. Data are expressed as mean ± SEM, *P  < 0.05. **c** Real-time PCR assay showed that RASAL1 expression was increased in the tumor tissues of RASAL1 overexpression group. Data are expressed as mean ± SEM, n = 4, *P < 0.05. **d** Western blotting assay showed that SCD1 expression was decreased in the tumor tissues of RASAL1 group. Data are expressed as mean ± SEM, n = 3, *P < 0.05

Western blotting analysis showed that G0/G1 phase cell cycle regulation protein expression was decreased in the HCT-116 cells of RASAL1 overexpression compared with that of control group in a dose-dependent manner, but show no effect on the G2/M and S phase regulating protein (Fig. 5a). These results were confused with the Fig. 2c, d, and the reason needs to be further verification. We next evaluated tumor growth of xenografts derived from HCT-116 cells that were transfected with vector control or RASLA1 by subcutaneous injection into nude mice. We found that tumor growth suppression was abrogated in colon cancer cells xenografts where RASAL1 was overexpressed (Fig. 5b). RT-qPCR and Western blot analysis showed that RASAL1 expression was increased in the tumor tissues of RASAL1 group compared with that of the control group, which decreased the SCD1 mRNA and protein levels (Fig. 5c, d). Taken together, we concluded that RASAL1 inhibited colon cancer cell proliferation in vitro and in vivo.

## Discussion

Colon cancer has become one of the most frequently occurring malignancies worldwide, but effective treatment options for colon cancer are limited [19]. Recently, RASAL1 has been shown to exert anti-neoplastic effects in many cancer cells including cancers of the gastric, oesophagogastric adenocarcinoma, thyroid and liver [5, 20–22].

As a negative modulator of the RAS signaling pathway by functioning as a Ras-GAP that catalyzes RAS inactivation, RASAL1 has been suggested to be a candidate tumor suppressor gene and impacts the profile of cancer in recent years [5]. However, direct evidence to demonstrate its tumor suppressor function is lacking. Previously studies found that, the increased expression of RASAL1 caused declined expression of RAS-GTP and p-ERK1/2, as well as promoted apoptosis and restrained cell proliferation, invasion and migration BGC-823 cells [23]. Transcriptional suppression of RASAL1 through aberrant promoter methylation contributes to endothelial to mesenchymal transition which was an important source of cancer-associated fibroblasts (CAFs), which are known to facilitate tumor progression in several ways [24]. RASAL1 also could inhibits HepG2 cell proliferation via HIF‑2α mediated gluconeogenesis [25].

To this end, we identified elevated mRNA levels of RASAL1 in tumor samples from patients with colon cancer, and evaluated the effects of RASAL1 on the behavior of human colon cancer cells and found that RASAL1 inhibited cell proliferation in LoVo and HCT-116 cells, and found that RASAL1 was in low expression levels in colon cancer tissues and cells, overexpression of RASAL1 inhibit the colon cancer cell proliferation, and the molecular mechanism was associated with the arrest of cell cycle signal transduction system. Indicating RASAL1 as a promising therapeutic target for the elimination of tumor-initiating colon cancer cells.

SCD1 is the rate-limiting enzyme in the synthesis of unsaturated fatty acids and responsible for a wide range of biological effects related to energy storage and signaling. Recently, it has been reported that SCD1 expression and activity are closely related to cancer pathogenesis and tumor malignancy, because tumor cells obtain most of fatty acid from de novo synthesis [26], and SCD1 inhibitors shows potential anticancer effect both in vivo and in vitro [13]. In view of the important role of lipid metabolism in colon cancer, we screened key regulator genes in lipid metabolism to comprehensively understand the effect of RASAL1 on colon cancer progression, and found that RASAL1 efficiently decreased protein expression of SCD1. Which was consistent with previous reports that SCD1 may be a tumor suppressor in colon cancer [27]. Furthermore, we demonstrated that RASAL1 inhibit the SCD1 activity via LXRα and SREBP1c pathway. Activation of LXRα with T0901317 or overexpression of SREBP1c both caused a significant up-regulation of SCD1 mRNA and protein levels. By co-transfecting SREBP1c with the SCD1–3′UTR reporter vector or co-treat with T0901317 in colon cancer cells, we detected the activity of luciferase to demonstrate the SREBP1c and LXRα targeting SCD1. At the same time, we made point mutations in SCD1–3′UTR to construct mutant SCD1–3′UTR fluorescence reporter plasmid. Both RASAL1 overexpression and SCD1 inhibitor A939572 decreased fluorescence activity and no significant changes in fluorescence activity were observed in mutant UTR.

In addition, overexpression of RASAL1 inhibited the cell cycle regulation protein expression in a dose-dependent manner and decreased tumor growth in xenograft nude mice models of colon cancer in vivo. We observed correlations between RASAL1 and SCD1 expression levels, suggesting that RASAL1 inhibits the cell proliferation by decrease the SCD1 activity.

## Conclusion

The data obtained in the present study suggest that the RASAL1/SCD1 axis might play an essential role in development of colon cancer cells, RASAL1 was able to inhibit the fatty acid synthesis by inhibit SCD1 3′-UTR activity via LXRα and SREBP1c pathway, then inhibit the cell proliferation by cell cycle arrest. Suggesting the signaling cohort could serve as a novel therapeutic target for the treatment of colon cancer.

## Data Availability

All data generated or analyzed during this study are included in this published article.

## Abbreviations

RASAL1: RAS protein activator like 1
Scd1: stearoyl-coenzyme A desaturase 1
LXRα: liver X receptor α
SREBF1: sterol regulatory element binding transcription factor 1
PPARγ: peroxisome proliferator activated receptor gamma
ACLY: ATP citrate lyase
FASN: fatty acid synthase
ACC: acetyl-CoA carboxylase
DGAT: diacylglycerol O-acyltransferase
MTTP: microsomal triglyceride transfer protein
ApoB: apolipoprotein B
VLDLR: very low density lipoprotein receptor
GAPDH: glyceraldehyde-3-phosphate dehydrogenase
